# Times of Regeneration

**Published:** 1920-04-17

**Authors:** 


### TIMES OF REGENERATION.

Lord Astor, who was one of the speakers at the Oxford Conference of the National Council of Social Service, spoke there, as he has spoken elsewhere, of the need for reorganisation among local public bodies. There is no doubt of it. A great number of localities have several public bodies dealing more or less with the same subject; and if the health service is to be thoroughly improved and developed, greater unification will be required. Take the case of Plymouth, for example. It has its Sanitary Committee, its Board of Guardians, Insurance Committee, Education Committee, and Port Sanitary Authority, each dealing with a portion of the health of the community. In some of the great industrial cities there is also a complete division and separation even within the City Council's own administration. The general health administration by the medical officer of health is frequently divorced from school medical work, and this does not appear to be a satisfactory plan. It is not easy, of course, to draw a distinct line of demarcation between health supervision as exercised through the Board of Education and its local committees on the one hand, and that of the Ministry of Health and the municipalities on the other. But the Ministry of Health ought to be the supreme authority on health, and the local medical officers ought to be the responsible executive officers for all health matters under the undivided control of the local councils. Health is too big a subject to be a subsidiary care of education authorities.

The multiplication of the local committees is due to the increasing burdens that have been thrust upon municipal bodies in the last ten or twenty years, and it is not easy to see how it could have been avoided. The wonder is that so many difficult tasks have been accomplished as well as they have, and it says much for the public spirit of the country. Viscount Astor's idea of the national aim is that every man, woman, and child should have available in every area, whether by fee or by payment, adequate diagnosis, nursing, treatment, or general help. This is a great programme, and it cannot be achieved without definite local centralisation. The pieces, however, are gradually being put together, and we are a considerable way along the road. The pessimist waxes cynical about the "new world," but its spirit nevertheless exists, and in no sphere is there greater opportunity or greater promise than in that of public health in all its ramifications. The times are difficult, but they are times of regeneration, bearing with them great new forces of women's social and political service such as have never previously existed in anything approaching the same degree.

